# Characterizing Industrial and Artisanal Fishing Vessel Catch Composition Using Environmental DNA and Satellite-Based Tracking Data

**DOI:** 10.3390/foods10061425

**Published:** 2021-06-19

**Authors:** Demian A. Willette, Gabriela Navarrete-Forero, Zachary Gold, Apollo Marco D. Lizano, Leonardo Gonzalez-Smith, Giovanna Sotil

**Affiliations:** 1 Biology Department, Loyola Marymount University, Los Angeles, CA 90045-2659, USA; lgonza52@lion.lmu.edu; 2 Centro del Agua y Desarrollo Sustentable, Escuela Superior Politécnica del Litoral, 090902 Guayaquil, Ecuador; gnavarr@espol.edu.ec; 3 Proyecto Redes Fantasma DAAD, Universidad San Francisco de Quito, 170901 Esmeraldas, Ecuador; 4 Department of Ecology and Evolutionary Biology, University of California, Los Angeles, CA 90095, USA; zack.j.gold@gmail.com; 5 Faculty of Bioscience & Aquaculture, Nord University, 8026 Bodø, Norway; marcopolo132004@gmail.com; 6 Laboratorio de Genética Molecular, Instituto del Mar del Perú-IMARPE, Callao 01, Lima, Peru; gsotil@imarpe.gob.pe

**Keywords:** environmental samples, commercial fishing, seafood, species identification, metabarcoding, traceability, tuna

## Abstract

The decline in wild-caught fisheries paired with increasing global seafood demand is pushing the need for seafood sustainability to the forefront of national and regional priorities. Validation of species identity is a crucial early step, yet conventional monitoring and surveillance tools are limited in their effectiveness because they are extremely time-consuming and require expertise in fish identification. DNA barcoding methods are a versatile tool for the genetic monitoring of wildlife products; however, they are also limited by requiring individual tissue samples from target specimens which may not always be possible given the speed and scale of seafood operations. To circumvent the need to individually sample organisms, we pilot an approach that uses forensic environmental DNA (eDNA) metabarcoding to profile fish species composition from the meltwater in fish holds on industrial and artisanal fishing vessels in Ecuador. Fish identified genetically as present were compared to target species reported by each vessel’s crew. Additionally, we contrasted the geographic range of identified species against the satellite-based fishing route data of industrial vessels to determine if identified species could be reasonably expected in the catch.

## 1. Introduction

Global per capita consumption of seafood now exceeds 20 kg [1], with rising demand driving substantial increases in fisheries exports from developing nations [2]. Seafood commands a value of USD 151 billion on the global market [1] and sustains the livelihood and nutrition of nearly 4.5 billion people [3]. The decline in wild-caught fisheries continues, however, and is pushing the need for improved management for long-term sustainability to the forefront of national and regional priorities. One major threat to sustainable wild-caught stocks is illegal, unreported, and unregulated (IUU) fishing activities. Emerging multilateral agreements by policy agencies including the Asia-Pacific Fishery Commission, the Southeast Asian Fisheries Development Center, the European Union, and the United States Seafood Import Monitoring Program are setting the framework for action on IUU fishing [4,5,6,7], namely the aim to develop personnel expertise and tools in accurate and cost-effective monitoring, control, and surveillance (MCS) of fisheries.

Ecuador is among the top 25 nations in marine capture production [8]. The industrial fishing fleet includes approximately 700 vessels using purse seine nets and longlines [9,10]. This fleet currently targets tropical tunas (e.g., *Katsuwonus pelamis*, *Thunnus albacares*, *Thunnus obesus*), common dolphinfish (*Coryphaena hippurus*), and Chilean jack mackerel (*Trachurus murphiyi*). In 2010, the industrial fleet produced 249,850 tons of fish, whereas the artisanal fleet produced 100,900 tons [11]. The artisanal fleet involves > 5500 fishing boats using lines and surface gillnets to catch the pelagic species *C. hippurus*, tropical tunas, billfish (e.g., *Xiphias gladius*), and sharks (e.g., *Alopias pelagicus*). Other common pieces of artisan gear are bottom gillnets for snappers (*Lutjanus* spp.), deep longlines for Pacific bearded brotula (*Brotula clarkae*) and several grouper species (*Epinephelus* spp.) locally known as murico or mero [12]. However, the pool of species that are caught by the artisanal fleet is megadiverse. For instance, more than 130 species have been recently recorded at two landing sites in the province of Esmeraldas, and most fish species are consumed locally (Navarrete-Forero, unpublished data). Commonly represented families include Carangidae, Clupeidae, Engraulidae, Haemulidae, Lutjanidae, Sciaenidae, and Serranidae.

Over the past 50 years, genetic technologies have made significant contributions to fisheries management and marine conservation [13,14,15]. DNA barcoding applications, for example, have highlighted the occurrence of and challenging circumstances associated with seafood mislabeling at all levels of the supply chain [16,17,18,19], including in certified seafood programs that emphasize traceability [20,21], and efforts to stem the illegal trade of protected species [22,23,24]. While barcoding methods are a versatile and quick tool for genetic monitoring of wildlife products [25], they do rely on obtaining tissue samples from harvested specimens, which may not always be possible given the scale, speed, and logistics of marine fishing operations and seafood processing.

Next-generation sequencing technology now permits the sequencing of residual DNA of target species indirectly from their surrounding environment, circumventing the need to capture and sample individual organisms (see reviews by [26,27,28]). Every living specimen sheds particles of genetic material in the form of scales, skin, hair, waste, etc., into their surrounding aquatic or terrestrial environment. Collectively, these are referred to as environmental DNA (eDNA). Metabarcoding allows for eDNA to be filtered from a representative sample of an environment and then sequenced to determine the species diversity of a location’s community [28,29]. Early development of eDNA metabarcoding methods successfully identified residents of large artificial mesocosms [30] with 93% accuracy [31]. In natural marine [32] and aquatic [33,34] habitats, not only have eDNA methods been able to identify typical species but they have also proven extremely useful at detecting species occurring at much lower abundances that would likely be missed with a traditional census survey [32,35]. Growth in eDNA approaches has been facilitated by initiatives such as the Tree of Life and Barcode of Life projects [25,36] that have compiled comprehensive and dynamic open access databases of genetic information, enabling rapid identification of species using standardized genetic “barcodes” [37]. While eDNA methods are a relatively new approach and efforts are underway to reduce uncertainty and improve accuracy in validating species presence, the realized benefits for fisheries include non-lethal assessments, early detection of invasive species, and cost effectiveness [27,32,38]. eDNA also holds promise for aiding in assessing fish biomass [39,40], screening for threatened species unintentionally corralled as fisheries by-catch [41], conducting gut content analysis [42], investigating holobiont/microbiome biodiversity [43], and tracking the frequency and impact of species that escape from aquaculture farms into open-water systems [44]. With growing national and international support to improve the MCS of fisheries, eDNA is an emerging tool that could be leveraged early in the seafood supply chain at points where monitoring has been previously limited or inaccessible.

Here, we pilot an approach that uses forensic eDNA metabarcoding to profile fish species composition from the meltwater in fish holds on industrial and artisanal fishing vessels. We apply a threshold criterion to enumerate and compare species-level identification among fishing vessel types to explore catch patterns. The genetic-based identification of present fish was compared to species catch composition reported by fishing vessel crew. Lastly, we traced the route of industrial vessel fishing activities using publicly available satellite-tracking data and contrasted the geographic range of identified species against a vessel’s fishing location to determine if the identified fish species could reasonably be expected in the catch. 

## 2. Materials and Methods

### 2.1. Field Sites and Vessel Sampling

**Figure 1:**
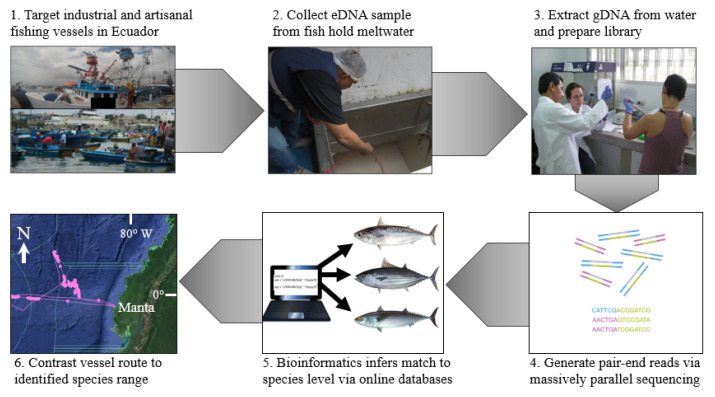
Schematic overview of experimental design using eDNA metabarcoding to characterize fish catch from industrial and artisanal fishing vessels in Ecuador.

Permission and access to sample from industrial and artisanal fishing vessels was coordinated and conducted with staff of the Instituto Público de Investigación de Acuicultura y Pesca (IPIAP) in collaboration with the Escuela Superior Politécnica del Litoral. Three industrial purse seine tuna fishing vessels (I_EC, I_DA, I_LZ) were sampled dockside at the Manta International Fish Port Complex (0°56′27″ S, 80°43′38″ W) on 14 July 2017, and three artisanal longline fishing vessels (A_AL, A_EC, A_ED) were sampled just prior to docking at the Facilidad Pesquera de Santa Rosa in Salinas (2°12′26″ S, 80°56′58″ W) on 17 July 2017 (Figure 1). The sampled vessels were chosen based on their presence in port, if they had been targeting tuna species, and the captain and crew’s willingness to allow sampling of meltwater. Upon boarding, the captain and crew were asked which fish species they targeted, the general location of their fishing activities, the type of gear used, and the trip’s duration, with all data confirmed for accuracy by accompanying IPIAP staff. Sampling from industrial vessels consisted of casting a 5 L pail tied to a rope into the pooled meltwater at the bottom of the fishing hold, dipping it up and down for 15 s, and then drawing it up to the deck to obtain three samples, which were stored in 50 mL conical tubes, sealed with Parafilm, and frozen for later processing. The smaller size of the artisanal vessels’ holds allowed for directly dipping the 50 mL tube into the pooled meltwater several times to mix and then remove the sample. Triplicate samples were taken from each vessel, labeled, and frozen until processing. 

### 2.2. eDNA Extraction and Library Preparation

eDNA was extracted and purified from sampled meltwater using a Qiagen DNeasy blood and tissue kit with modifications ([45], see Supplemental Materials S1 for protocol details). Preparation of sequencing libraries for eDNA analysis consisted of a two-step polymerase chain reaction protocol [46]. All reactions were conducted in PCR tubes using commercially available reagents (see Supplemental Materials S2 for product and protocol details). The first PCR treatment used non-indexed primers with Illumina Nextera adapter sequences, genomic DNA template, and Qiagen multiplex master mix; and the second PCR treatment used a template from the first PCR, and unique combinations of Illumina Nextera indexing primers. All amplifications were performed using Kapa high-fidelity polymerase master mix. In the first PCR, an assortment of universal and taxon-specific primers were available for use and are detailed in Supplemental Materials S2. Here, the universal MiFish-U primers [31], which were designed from a short hypervariable region (163–185 bp) of the 12S rRNA gene, were used to screen from hundreds of marine fish species. During the second PCR, unique combinations of indexing primers were added to each individual sample, thus allowing all uniquely indexed samples to be combined in equal concentrations, in a single sequencing reaction on the Illumina HiSeq 2500 platform, and distinguished during the bioinformatics stage. Prepared libraries were processed in a single lane on an Illumina HiSeq 2500 platform using paired-end sequencing.

### 2.3. Read Processing and Bioinformatics

Raw reads were processed using the modular metabarcode sequence toolkit *Anacapa* (available online: http://github.com/limey-bean/Anacapa/ (accessed on 16 May 2021)) [47] and the contained R-based package, *ranacapa* [48]. Generated paired-end sequencing reads were processed through the *Toolkit*’s quality control and ASV (amplicon sequence variant) parsing module to trim, filter out singletons and low-quality reads, and ultimately return ASV FASTA files and ASV count summary tables. These files were then processed through the *Toolkit*’s *Anacapa Classifier* module to assign taxonomy, where ASVs were queried against the *CRUX-generated 12S* reference database. Taxonomic identity was assigned probabilistically using the *Toolkit*’s modified Bayesian Least Common Ancestor method using a cutoff level of 60, following [46]. Furthermore, given uneven sequencing depth across samples, taxa with less than 1% of the proportion of reads were excluded from the analysis. 

### 2.4. Pairing Industrial Vessel Route and Metabarcoding Results

To further validate the taxonomic accuracy of species identified from meltwater samples, we contrasted industrial fishing vessels’ travel routes against the known geographic distribution of identified fish species. This was achieved by retrieving data from the ship’s automatic ship identification system (AIS) which publicly broadcasts its identity, position, and course while at sea. AIS was originally conceived to prevent at-sea ship collisions, yet has gained attention as a powerful tool to monitor fishing vessels with that mass detection of AIS via satellite (S-AIS) [49,50]. Here, we recorded the name, Maritime Mobile Service Identity (MMSI), and flag state for the sampled industrial fishing vessel in Manta, Ecuador. We then used the public database maintained by Global Fishing Watch (available online: http://globalfishingwatch.org (accessed on 20 February 2021) to retrieve the vessel’s travel route prior to making port on our day of sampling. Instances of fishing were inferred from locations where vessel speeds slowed below 4 knots and movement occurred in a “zig-zag” pattern [51,52]. We used computer-generated native distribution maps by AquaMaps [53] to cross reference the geographic range of identified fish to see if the known range overlapped with the vessel’s fishing location.

## 3. Results

A total of 2,588,151 12S paired-end sequencing reads were generated from the meltwater samples from the three industrial and three artisanal fishing vessels. After applying the 1% cutoff threshold at the sample level, reads were matched to six taxonomic orders within the class Actinopterygii. These were further delimited into eight families, 13 genera, and 12 species (Table 1). Of the total reads (post-cutoff), 72.4% (565,484/781,086) were matched at the species level.

Of the 12 identified species, no single species was found across all sampled industrial and artisanal vessels (Table 2). Skipjack tuna *K. pelamis* and a genus-level match to tuna, *Thunnus* spp., were identified in samples from all three industrial vessels, whereas no species was commonly identified across the three artisanal vessels. In contrast, nine of the 12 identified species were unique to a single fishing vessel. A similar number of distinct fish species were genetically identified among the artisanal and industrial fishing vessels (one to four species per vessel) (Table 2). 

No species reported as a target fish by any artisanal vessel was also identified in the eDNA results (Table 3). Notably, the target fish *Merluccius gayi* of the vessel A_AL was not found but the congener *Merluccius productus* was identified. Two GenBank entries for *M. gayi* (Accession FJ215054, DQ274005) did not match. There are, however, sequences for *T. alalunga* available on GenBank and this species was targeted by all sampled vessels, yet *Thunnus* cannot be resolved here and requires other primer sets. In contrast, on industrial vessels, two fish species, *Acanthocybium solandri* and *K. pelamis*, were reported by crew and identified genetically.

**Figure 2:**
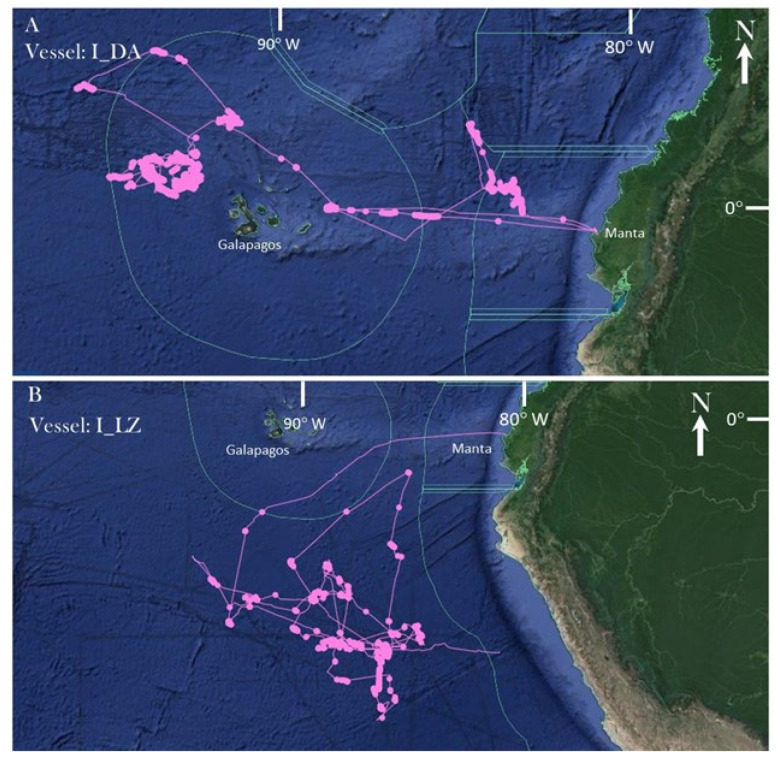
Fishing route (pink line) of industrial fishing vessels (**A**) I_DA and (**B**) I_LZ based on AIS information. Fishing duration was 35 and 76 days, respectively. Green line indicates Economic Exclusive Zones of 200 nautical miles from shore. Imagery adapted from GlobalFishingWatch.org.

The route of the representative fishing vessel I_DA was traced for the 35-day fishing voyage and of I_LZ for a 76-day fishing voyage prior to making port in Manta, Ecuador on the sampling day based on AIS data obtained from the Global Fishing Watch database (Figure 2). Both vessels’ routes coincided with the verbal description and duration provided by the crew on the sampling day. The two species-level and one genus-level matches from the I_DA eDNA samples (*Auxis thazard*, *K. pelamis*, and *Thunnus* sp.) all had a high (0.80–1.00) relative probability of occurrence in the regions where fishing activity likely occurred (as indicated by “zig-zag” movement of the vessel) per each species’ computer-generated native distribution maps [33] (Figure 2A). Likewise, all four species-level and one genus-level match from the I_LZ eDNA sample had high (0.80–1.00) relative probability of occurrence where fishing took place. AIS data for I_EC was unavailable on the Global Fishing Watch database, whereas Ecuadorian artisanal vessels are not equipped nor required to transmit AIS data. 

## 4. Discussion

Here, we have demonstrated that eDNA barcoding can be employed to characterize the species composition of captured fish from the meltwater leftover in industrial and artisanal fishing operations. eDNA is gaining popularity and utility in fisheries and seafood research and has the potential to be a potent and complementary tool to the growing number of approaches to improving traceability throughout the seafood supply chain [38,40,54,55]. Built upon nearly two decades of studies applying the well-vetted concept of DNA barcoding to identify fish to species level [16,56], the eDNA approach is positioned to be both a familiar and a transformative tool in improving seafood monitoring. Yet, the breadth, scale, and variety of global marine fisheries has and will continue to require an assortment of strategies to validate species identity.

Although there is room for further development of this study’s methods to achieve greater agreement between the reported target species and those species identified from the eDNA samples, our findings are encouraging and reflect a shortcoming of the current pilot study and do not represent an indictment on metabarcoding approaches themselves. For example, eDNA approaches failed to identify South Pacific hake *Merluccius gayi*, yet the congener North Pacific hake *M. productus* was identified. This was driven by a lack of 12S reference barcode sequence for this fisheries target. It highlights the importance of accurate and complete databases for successful applications of eDNA metabarcoding approaches, mainly on those markers frequently used for species identification. The Barcode of Life and recent efforts to generate region-specific reference databases such as Mo’orea BIOCODE [57] and California Current Large Marine Ecosystem [58] have dramatically improved the taxonomic assignment capabilities of eDNA metabarcoding. Thus, for the successful application of metabarcoding approaches for MCS, regional reference databases of priority species should be barcoded, allowing for tailored assays and improved resolution. 

Our study design targeted vessels fishing tuna and eDNA sampling did detect *Thunnus* present from four vessels, including all three industrial vessels. We were, however, only able to resolve genus-level matches for tunas (*Thunnus* sp.). These results are not surprising as previous research has demonstrated that the MiFish Universal Teleost primers used here do not capture sufficient genetic variation to resolve this recent adaptive radiation. Thus, future applications of eDNA metabarcoding that require species-level resolution of *Thunnus* will need to use alternative markers such as the *Thunnus*-specific primer [31]. This highlights the need for thoughtful consideration into primer design and benchmarking to adequately address specific monitoring questions of interest, particularly if these assays will be used for enforcement. Notably, the common Spanish name, albacora, reported by crew can also be problematic as it regionally refers to *T. alalunga*, as well as *T. albacares* and *T. obesus* [59]. We note that similar eDNA metabarcoding approaches could be used for a broad suite of fisheries targets, including cryptic and hard-to-identify species, as well as invertebrate targets, employing different genetic markers tailored for specific targets. Despite these minor limitations observed here, our results demonstrate that even “universal” teleost primer sets can provide important traceability information.

Sampled industrial fishing vessels reported more targeted fish species (six) than artisanal vessels (three) but were found to have similar numbers of species genetically identified (seven) as present (Table 2 and Table 3). Differences between industrial and artisanal catches might be attributed to several factors, including fishing effort, fishing duration, and fishing gear (pelagic longline versus purse seiner). All taxa reported by industrial vessel crew and identified genetically in the eDNA samples had known geographic ranges in the eastern Pacific, including the Galapagos Islands. Furthermore, some species identified genetically may not have been targeted fish, yet were still incidentally captured. Examples include *A. rochei* and *A. thazard* that can be also captured in purse seiner nets, *E. volitans* which is prey of tuna species, and reef species *C. maculata* which may also be found off the shore of oceanic islands [59]. Furthermore, fishers normally report commercial species as targeted because they fetch the best price, yet they usually catch many other species of lower economic value which are shared with crew members, port assistants, and relatives [60]. It may be that some species identified in the eDNA samples were not precisely by-catch and are important items for food security for coastal communities [60].

One of the limitations of this study is that the true fish species within each hold were unknown. For example, although the crew of the three artisanal fishing vessels cited targeting three species for capture, seven non-target species were identified genetically (Table 2 and Table 3). These data may provide insight into which species may be taken accidentally as by-catch, an area of research in artisanal fishing that has largely been overlooked [61]. However, given the high sensitivity of these molecular methods, it is unknown whether DNA from any of these species may have persisted from previous fishing excursions or been transferred by other sources (tracked in on boots, on the ice within the holds, etc.) as fishing vessels are hardly sterile environments. Furthermore, the industrial fishing vessels had multiple holds and the failed detection of some species may be a result of not sampling every hold aboard the active vessel. It is possible we sampled a given hold filled with catch from a certain schedule (e.g., afternoon), whereas dorado (*C. hippurus*) was captured in the morning and stored in another hold.

Likewise, these data cannot determine if a species is absent from the fish hold. In the case of *A. solandri*, *C. hippurus*, and *E. lineatus* being reported as target fish by the crew of I_DA, and these fish occurring where fishing activity occurred, we did detect their signature in the genetic sample, but far below the 1% cutoff threshold used to confirm presence. A reason why may have been related to the preservation of eDNA particles in aquatic environments, which are believed to be best preserved when conditions are alkaline and kept out of solar radiation exposure [62,63]. Cooler temperatures also prolong the viability of eDNA, lasting 10 days at 25 °C and up to 53 days at 5 °C [63]. However, the volume to detect species of interest may also be a limitation. For example, [64] suggested a sample volume of 100 L to ensure a high probability of capturing eDNA from aquatic environments with a density of fish of 0.32 g m^2^. Here, we were less concerned with obtaining enough eDNA particles overall, given the high concentration of biomass in the fish holds, versus the greater concern that the collected sample was a representative sample of the whole hold due to the high level of fish mucus and scales in the melt water. Future work could get around this issue by pooling multiple samples collected from around the fish hold to maximize probability of detection.

Lastly, we were unable to identify species relative abundance or biomass from a catch. The ability to use eDNA metabarcoding for abundance or biomass estimation remains equivocal [65], although recent applications appear promising [40,66]. Importantly, theoretical advancement in our understanding of PCR bias suggests that the application of mock communities can be strategically used to allow for the correction and estimation of abundance [67,68]. However, more work is needed to demonstrate the ability of eDNA metabarcoding approaches to provide meaningful abundance estimates [69], particularly in fish hold settings as explored here.

## 5. Conclusions

Overall, the main target species of tuna (*K. pelamis*, *Thunnus* spp.) were both reported by crew and identified in the meltwater eDNA samples for all industrial vessels. Results were less consistent for artisanal vessels. Furthermore, nine of 12 genetically identified species were unique to a single fishing vessel, providing insight into the diversity of captured fishes. Genetic tools, including eDNA metabarcoding, are well positioned to aid in the monitoring and management of seafood throughout the supply chain. Arguably, we no longer need to continue demonstrating the potential of genetics but now must accelerate its implementation [70]. For example, advancements in the application of eDNA for population genetics holds promise to elucidate geographic origin and stock structure of fisheries targets [71]. To date, there have been successful applications of eDNA to identify unique haplotypes associated with specific stock structures and geographic location [72,73,74,75]. As these methods improve, such applications could provide critical information for not only identifying species from fishing holds, but also stocks and likely geographic origin [40,76], aiding in the management and traceability of fishing fleets. To meet the objectives of national and multinational fisheries policies to improve seafood traceability and combat IUU fishing, transformative action should aim to (a) apply the most appropriate technologies that are also portable and accessible [77] and (b) build a monitoring and enforcement capacity that spans from fisher to consumer [5].

## Figures and Tables

**Table 1:** Taxonomic classification of amplicon sequence variants (ASVs) across all sampled fishing vessels. ““ indicates same taxonomic level as above cell. -indicates no data.

Order	Family	Genus	Species (Common Name)
Acanthuriformes	Scianenidae	-	-
Beloniformes	Exocoetidae	*Exocoetus*	*Exocoetus volitans* (two-wing flying fish)
““	Hemiramphidae	*Oxyporhamphus*	*Oxyporhamphus Micropterus* (bigwing halfbeak)
Carangiformes	Carangidae	*Seriola*	*Seriola rivoliana* (longfin yellowtail)
““	““	*Chloroscombrus*	*Chloroscombrus orquesta* (Pacific bumper)
““	““	*Decapterus*	*Decapterus macrosoma* (shortfin scad)
Gadiformes	Merlucciidae	*Merluccius*	*Merluccius productus* (North Pacific hake)
Istiophoriformes	Istiophridae	*-*	*-*
Scombriformes	Scombridae	*Acanthocybium*	*Acanthocybium solandri* (wahoo)
““	““	*Auxis*	*Auxis rochei* (bullet tuna)
““	““	““	*Auxis thazard* (frigate tuna)
““	““	*Katsuwonus*	*Katsuwonus pelamis* (skipjack tuna)
““	““	*Sarda*	*Sarda orientalis* (striped bonito)
““	““	*Euthynnus*	*-*
““	““	*Thunnus*	*-*
Tetradontiformes	Balistidae	*Canthidermis*	*Canthidermis maculate* (rough triggerfish)

**Table 2:** Species- or genus-level taxa identified in eDNA samples as present (P) in artisanal and industrial fishing vessels. Grey highlighting signifies taxon was listed by crew/captain as targeted fish in catch.

	Artisanal Fishing Vessel			Industrial Fishing Vessel		
Species	A_AL	A_EC	A_ED	I_DA	I_EC	I_LZ
*Acanthocybium solandri*						P
*Auxis rochei*		P				P
*Auxis thazard*		P		P	P	
*Canthidermis maculate*					P	
*Chloroscombrus sorqueta*	P					
*Decapterus macrosoma*		P				
*Exocoetus volitans*						P
*Katsuwonus pelamis*				P	P	P
*Merluccius productus*	P					
*Oxyporhamphus Micropterus*					P	
*Sarda orientalis*		P				
*Seriola rivoliana*			P			
**Genus level only**						
*Euthynnus*					P	
*Thunnus*			P	P	P	P

**Table 3:** Species reported by each vessel’s crew as targeted fish (P) in catch. Spanish name reported in parenthesis. Peje sierra is also sometimes used for *Scomberomorus sierra* and albacora may sometimes be used for *Thunnus albacares* and *T. obesus* regionally.

Species	A_AL	A_EC	A_ED	I_DA	I_EC	I_LZ
*Acanthocybium solandri* (peje sierra)				P		P
*Coryphaena hippurus* (dorado)				P		P
*Euthynnus lineatus* (pata seca)				P		
*Istiompax indica* (picudo)						P
*Katsuwonus pelamis* (bonito)		P		P	P	P
*Merluccius gayi* (merluza)	P					
*Thunnus alalunga* (albacora)	P	P	P	P	P	P

## Data Availability

The data presented in this study are available within the article and on request from the corresponding author.

## Supplementary Materials

The following are available online at https://www.mdpi.com/article/10.3390/foods10061425/s1, S1: eDNA extraction protocol, S2: Library preparation protocol.
