# Supplementary material for: Characterizing Industrial and Artisanal Fishing Vessel Catch Composition Using Environmental DNA and Satellite-Based Tracking Data
Source: Foods. 2021 Jun 19;10(6):1425. doi: 10.3390/foods10061425 (PMC8235475; doi:10.3390/foods10061425)
Supplement: Supplementary file 1 [file foods-10-01425-s001.zip › foods-1254812-supplementary.pdf]

Supplemental Materials 1 – eDNA extraction
Willette et al. Characterizing commercial and artisanal fishing vessel catch composition using environmental DNA and satellite-based tracking data

Overview

Below instructions are provide for purification of eDNA from Sterivex filters, and extraction of eDNA using Qiagen DNeasy blood and tissue kit. Instructions assume water containing target eDNA have been screened through a 0.22µm Sterivex filter (Millipore Corp.), and samples have been kept chilled or frozen from the time sampled to the beginning of this protocol. Additionally, it is critical that all sampling was conducted using sterile equipment and efforts were made to eliminate cross-contamination with non-target eDNA. In most cases, the use of both 10% bleach solution and >70% ethanol effectively sterilize surfaces.

Day 1: Lysing of eDNA sample

Materials

eDNA samples in Sterivex filters with Luer Lock caps on
1000 µL pipette with sterile tips
200 µL pipette with sterile tips
10% bleach solution (in spray bottle)
>70% ethanol (in spray bottle)
Kimwipes
Proteinase K
Qiagen ATL buffer
Rotating incubator

Methods

1. Turn on rotating incubator, set a temperature of 56°C.
2. Begin by spraying work surface with bleach, wipe clean with Kimwipe, spray with ethanol, and wipe clean with Kimwipe.
3. Remove Luer cap from the female end of the Sterivex filter.
4. Slowly pipette 720 µL of ATL buffer directly on top of the filter media through the female end opening (avoid backsplash by expelling slowly).
5. Slowly pipette 80 µL of Proteinase K directly on top of the filter through the female opening, again avoiding backsplash.
6. Firmly secure a new, sterile Luer cap over the female end, and wrap Parafilm around cap.
7. Repeat for all eDNA samples.
8. Place Sterivex filters in the rotating incubator, turn on rotating function so that the solution gently washes back and forth over the entire filter. Allow filters to incubate overnight/for a minimum of 12 hours.

Note on Proteinase K - Proteinase K is a serine protease, a broad-spectrum enzyme that cleaves peptide bonds in proteins (including in hair or keratin, hence the 'K'). Proteinase K is stable at a wide pH range (optimum pH 8.0, stable at pH 4-12), may be stored at room temperature (15-25°C), and has an elevated activity at 50-60°C. Proteinase K used with a buffer, in our case we will use the Qiagen tissue lysis buffer ATL. Proteinase K and ATL may cause mild skin irritation; wear protective gloves when handling.

Day 2: Extraction of eDNA sample using Qiagen DNeasy kit

Materials

eDNA samples in Sterivex filters, after at least 12 hours Proteinase K/ATL incubation
10% bleach solution (in spray bottle)
>70% ethanol (in spray bottle)
Kimwipes
1000 µL pipette with sterile tips
200 µL pipette with sterile tips
Qiagen DNeasy Tissue and Blood kit, one prep per sample
2 pre-labeled 1.5 mL LoBind Eppendorf tubes per sample
50 mL tube with molecular grade ethanol (99 or 100%), in freezer or chilled on ice
Aliquot of AE buffer (from kit) heated to 70°C. Aliquot 100 µL per sample + extra 200 µL
Vortex and centrifuge

Pre-Methods

1. Place 50 mL tube of ethanol in freezer or on ice to chill, will use later
2. Heat aliquot of AE buffer on heating block at 70°C
3. Make sure two sets of 1.5 mL tubes are pre-labeled

Methods

1. Sterilize all work surfaces with 10% bleach and ethanol.
2. Obtain samples from rotating incubator.
3. Remove the female and male end caps from filter, gently remove all liquid from filter using a 3 mL sterile syringe.
4. Transfer liquid to a 1.5 mL Eppendorf tube. **Maximum volume per tube can only be 450 µL. If you total volume is greater than 450 µL, use as many tubes as needed. In some cases, 2-3 tubes may be needed.
5. Measure the volume of liquid to the nearest 10 µL using pipette. Record this volume, per tube/sample, on a sheet of paper.
6. Mix equal amounts of sample, AL buffer, and ice-cold ethanol. Example, for a tube with 450 µL of sample, add 450 µL of each AL buffer and ethanol for a total volume of 1,350 µL (or 1.35 mL). Repeat for all tubes for each eDNA sample.
7. Vortex tube vigorously for 10 seconds.
8. Transfer 750 µL of the liquid to a labeled Qiagen spin column.
9. Centrifuge at 8,000 rpm for 1 minute.
10. Discard flow-through, and dab the rim of the spin column dry on a Kimwipe.
11. Repeat steps 8-10 until all the liquid from the eDNA sample has been spun through the spin column (may be 5+ times depending on initial liquid from Sterivex).
12. Place the DNeasy spin column in a new 2 mL collection tube (provided in kit)
13. Add 500 µL of Buffer AW 1 directly to the spin column filter (provided in kit)
14. Centrifuge for 1 minute at 8,000 rpm.
15. Discard flow-through, and the collection tube.
16. Place the DNeasy spin column in a new 2 mL collection tube (provided in kit)
17. Add 500 µL of Buffer AW 2 directly to the spin column filter (provided in kit)
18. Centrifuge for 3 minutes at 14,000 rpm. Spin column should appear dry.
19. Discard flow-through and dab rim of spin column on a clean Kimwipe.
20. Place the spin column back in collection tube, centrifuge for 1 minute at 14,000 rpm.
21. Remove spin column from collection tube and place column in a new, pre-labeled 1.5 mL Eppendorf tube. The cap should be left open.
22. Add 100 µL of pre-heated (70°C) AE buffer directly onto the center of the spin column filter.

23. Incubate at room temperature for 7 minutes.
24. Centrifuge for 1 minute at 8,000 rpm to release/elute DNA extract into tube.
25. Repeat elution once with the flow-through now in the collection tube.
26. Transfer all elute into a new, pre-labeled 1.5 mL tube. Close tightly.
27. Confirm label is accurate and complete. Includes sample location, date, etc.
28. Parafilm the tube to secure cap.
29. Store at -20C for a minimum of 8 hours. May be stored overnight.
30. Your sample is now ready for PCR.

Supplemental Materials 2 - Preparing eDNA sequencing libraries
Willette et al. Characterizing commercial and artisanal fishing vessel catch composition using environmental DNA and satellite-based tracking data

Overview

Preparation of sequencing libraries for eDNA analysis consists of treating genomic DNA with two sets of polymerase chain reactions. All reactions are conducted in PCR tubes using commercially available reagents (see product information/numbers below).

In this double PCR method, the first PCR treatment uses non-indexed primers, genomic DNA template, and the Qiagen master mix; and the second PCR treatment uses template from the first PCR, Nextera indexing primers, and HiFi polymerase master mix.

During the second PCR, unique combinations of indexing i5 and i7 primers (e.g. N501 + N701) are added to each individual sample, thus allowing all samples, now uniquely indexed, to be combined in a single sequencing lane and distinguished during the bioinformatics stage.

As done in the eDNA extraction protocol, it is critical that laboratory work be done using sterile equipment and efforts were made to eliminate cross-contamination with non-target eDNA. In most cases, the use of both 10% bleach solution and >70% ethanol effectively sterilize surfaces.

Step 1: First PCR with non-indexed primers

Materials

High quality gDNA from eDNA extractions
10% bleach solution (in spray bottle)
>70% ethanol (in spray bottle)
1-10 μL pipette with sterile tips
10-200 μL pipette with sterile tips
0.6 mL PCR tubes
Qiagen multiplex master mix (Qiagen, #206143)
Primers
Molecular grade water
Thermal cycler, centrifuge
1% agarose gel, gel electrophoresis set-up

Methods

9. Enter/check accuracy of the touchdown PCR protocol on thermal cycler
10. Sterilize all work surfaces with 10% bleach and ethanol.
11. Prepare the following master mix for PCR. This recipe is for a single reaction with two sets of primers (miFish and MiCOI), please scale up as needed.

Qiagen 2x Master Mix	12.5 μL
Primer MiFish-U-F (2μM)	2.5 μL
Primer MiFish-U-R (2μM)	2.5 μL
Primer MiCOI-lintF (2μM)	2.5 μL
Primer MiCOI-lintR (2μM)	2.5 μL
ddH_20	1.5 μL

12. Aliquot 24 μL of the prepared master mix into individually labeled 0.6 mL PCR tubes.
13. Add 1 μL of gDNA template to each tube accordingly. Gently back pipette to mix.
14. Repeat for all samples. Briefly centrifuge to assure mix is at bottom of tube.
15. Place tubes on thermal cycler and begin the touchdown PCR protocol.
16. Remove tubes from thermal cycler and keep on ice.
17. Check sample for successful amplification using gel electrophoresis. You may use 3-5 μL for this step. The target size of the product is between 163bp and 185bp, thus use a 2% gel at 100V for at least 20 minutes, run with an appropriate ladder.
18. Keep successful product at -20°C until ready to proceed to next step.

Note on primers for first PCR – An assortment of universal and taxon-specific primers are available for use in this method. Here we are using the universal MiFish-U primers (Miya et al. 2015), which was designed from a short hypervariable region (163-185bp) of the 12S rRNA gene to detect more than 230 marine fish species. Also available are the MiFish-E primers, designed to identify elasmobranchs (sharks and rays) for which the MiFish-U primer fails to do well. The MiFish-E primers amplify a 170-185 bp region of the 12SrRNA gene region. The MiFish-tuna primers target seven tuna species (genus Thunnus) that the MiFish-U also fails to identify well. The MiFish-tuna primers amplify a 180bp portion of the ND5 gene. Lastly, miCOI degenerate COI primers target a broad range of metazoan species from the marine environment including many invertebrate species.

Step 2: PCR clean-up with ExoSAP-IT

Materials

PCR product from first PCR
ExoSAP-IT (Fisher #78200.20.UL)
1-10 μL pipette with sterile tips
0.6 mL PCR tubes
Molecular grade water
10% bleach solution (in spray bottle)
>70% ethanol (in spray bottle)
Thermal cycler, centrifuge

Methods

1. Remove ExoSAP-IT from freezer and keep on ice during use.
2. Mix 2 μL of ExoSAP-IT reagent to every 5 μL of PCR product. After the gel check, you should still have ~20 μL of product available, thus add 8 μL of ExoSAP-IT to first PCR tube. Back pipette to mix, and briefly centrifuge tube. Total volume will be ~28-30 μL.

3. Using thermal cycler incubate tubes at 37°C for 15 minutes, followed by 80°C for 15 minutes to inactivate ExoSAP-IT reagent.
4. Keep samples/tubes at -20°C until ready to proceed to next step.

Step 3: Second PCR with indexing primers

Materials

PCR product from first PCR
Nextera Indexing kit (Illumina # FC-121-1011)
High fidelity polymerase master mix (KAPA HiFi HotStart, Fisher # NC0295239)
1-10 μL pipette with sterile tips
10-200 μL pipette with sterile tips
0.6 mL PCR tubes
Molecular grade water
10% bleach solution (in spray bottle)
>70% ethanol (in spray bottle)
Thermal cycler, centrifuge

Methods

1. Enter/check accuracy of the Indexing PCR protocol on thermal cycler
2. Sterilize all work surfaces with 10% bleach and ethanol.
3. Prepare the following master mix for second PCR. This recipe is for a single reaction at a total volume of 50 μL. *** **Note – Each sample must have unique pair of i5 and i7 primers**. For our purposes, we will prepare PCR tubes individually to assure assignment of unique indexes for each sample.

Molecular grade water	12.5 μL
HiFi polymerase master mix	25 μL
Primer i5 (unique per sample)	1.25 μL
Primer i7 (unique per sample)	1.25 μL
PCR product (~10 ng)	10 μL

4. Remove reagents from freezer and keep on ice.
5. In individually labeled 0.6 μL, aliquot reagents, starting with water, to each tube.
6. *****Be certain each tube receives a unique pair of i5 and i7 primers, record assigned primers to each sample in your notebook/worksheet. E.g. 'Manta 1, N502, N701'**
7. Gently back pipette to mix.
8. Repeat for all samples. Briefly centrifuge to assure mix is at bottom of tube.
9. Place tubes on thermal cycler and begin the Indexing PCR protocol.
10. Remove tubes from thermal cycler.
11. Check sample for successful amplification using gel electrophoresis. You use 3 μL for this step. The target size of the product is ~260bp, thus use a 2% gel at 100V for at least 20 minutes, run with an appropriate ladder.

Oligonucleotide sequences

Name	Sequence	Target Taxa	Source
MiFish-U-F	TCG TCG GCA GCG TCA GAT GTG TAT AAG AGA CAG GTG TCG GTA AAA CTC GTG CCA GC	+230 subtropical marine fish species	Miya et al. 2015
MiFish-U- R	GTC TCG TGG GCT CGG AGA TGT GTA TAA GAG ACA GCA TAG TGG GGT ATC TAA TCC CAG TTT G		Miya et al. 2015
MiFIsh-E - F	TCG TCG GCA GCG TCA GAT GTG TAT AAG AGA CAG GTG TTG GTA AAT CTC GTG CCA GC	Elasmobranchs (sharks and rays)	Miya et al. 2015
MiFIsh-E - R	GTC TCG TGG GCT CGG AGA TGT GTA TAA GAG ACA GCA TAG TGG GGT ATC TAA TCC TAG TTT G		Miya et al. 2015
MiFish-tuna-ND5-F	ACA CTC TTT CCC TAC ACG ACG CTC TTC CGA TCT NNN NNN ATG TCC TTC CTC CTT ATC GGC TG	*Thunnus* species (tuna)	Miya et al. 2015
MiFish-tuna-ND5-R	GTG ACT GGA GTT CAG ACG TGT GCT CTT CCG ATC TNN NNN NTT GCC AGT GGC AGC TAC GAT C		Miya et al. 2015
mlCOIintF	GGW ACW GGW TGA ACW GTW TAY CCY CC	Broad range of metazoans	Leray et al. 2013
mlCOIintR	GGG RGG RTA SAC SGT TCA SCC SGT SCC		Leray et al. 2013

1st PCR reaction – Non-indexing PCR protocol

Step	Temperature (°C)	Time	Cycles
Initial denature	95°C	5 minutes	1
Denature	94°C	30 seconds	Anneal – Start at 69.5°C, decrease 1.5°C per cycle for 13 cycles, 13th cycle at 50°C.
Anneal	VARIABLE	30 seconds	
Elongate	72°C	90 seconds	
Denature	94°C	30 seconds	Repeat for 25 cycles
Anneal	50°C	30 seconds	
Elongate	72°C	45 seconds	
Final elongate	72°C	10 minutes	1
Hold	4°C	Forever	

2nd PCR reaction - Indexing PCR protocol

Step	Temperature (°C)	Time	Cycles
Initial denature	95°C	5 minutes	1
Denature	98°C	20 seconds	Repeat for five (5) cycles (if conc. <5ng, use 12 cycles)
Anneal	56°C	30 seconds	
Elongate	72°C	3 minutes	
Final elongate	72°C	5 minutes	1
Hold	8°C	Forever	
